# 1-[4-(2-Chloro­eth­oxy)-2-hy­droxy­phen­yl]ethanone

**DOI:** 10.1107/S1600536810054632

**Published:** 2011-01-22

**Authors:** Li Wang, Shuo Jiao, Peng Shi, Ge-Hong Wei

**Affiliations:** a College of Life Sciences, Northwest A&F University, Yangling Shaanxi 712100, People’s Republic of China

## Abstract

In the title compound, C_10_H_11_ClO_3_, obtained by the reaction of 2,4-dihy­droxy­acetophenone, potassium carbonate and 1-bromo-2-chloro­ethane, an intra­molecular O—H⋯O hydrogen bond occurs.

## Related literature

The title compound was synthesized by the Williamson reaction (Dermer, 1934 ▶). For a related structure, see: Schlemper (1986 ▶).
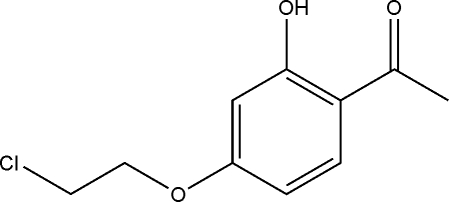


## Experimental

### 

#### Crystal data

C_10_H_11_ClO_3_
*M* *_r_* = 214.64
Orthorhombic, 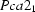

*a* = 8.9970 (7) Å
*b* = 5.3258 (4) Å
*c* = 20.6307 (17) Å
*V* = 988.55 (13) Å^3^
*Z* = 4
Mo *K*α radiation
μ = 0.36 mm^−1^
*T* = 298 K
0.40 × 0.39 × 0.20 mm

#### Data collection

Siemens SMART CCD area-detector diffractometer
Absorption correction: multi-scan (*SADABS*; Sheldrick, 1996 ▶) *T*_min_ = 0.868, *T*_max_ = 0.931
4434 measured reflections
1664 independent reflections
1121 reflections with *I* > 2σ(*I*)
*R*_int_ = 0.063

#### Refinement

*R*[*F* ^2^ > 2σ(*F* ^2^)] = 0.052
*wR*(*F* ^2^) = 0.141
*S* = 1.07
1664 reflections
127 parameters
1 restraint
H-atom parameters constrained
Δρ_max_ = 0.24 e Å^−3^
Δρ_min_ = −0.21 e Å^−3^
Absolute structure: Flack (1983 ▶), 758 Friedel pairs
Flack parameter: 0.10 (14)

### 

Data collection: *SMART* (Siemens, 1996 ▶); cell refinement: *SAINT* (Siemens, 1996 ▶); data reduction: *SAINT*; program(s) used to solve structure: *SHELXS97* (Sheldrick, 2008 ▶); program(s) used to refine structure: *SHELXL97* (Sheldrick, 2008 ▶); molecular graphics: *SHELXTL* (Sheldrick, 2008 ▶); software used to prepare material for publication: *SHELXTL*.

## Supplementary Material

Crystal structure: contains datablocks I, global. DOI: 10.1107/S1600536810054632/jh2251sup1.cif

Structure factors: contains datablocks I. DOI: 10.1107/S1600536810054632/jh2251Isup2.hkl

Additional supplementary materials:  crystallographic information; 3D view; checkCIF report

## Figures and Tables

**Table 1:** Hydrogen-bond geometry (Å, °)

*D*—H⋯*A*	*D*—H	H⋯*A*	*D*⋯*A*	*D*—H⋯*A*
O2—H2⋯O1	0.82	1.81	2.533 (5)	145

**Fig. 1.**
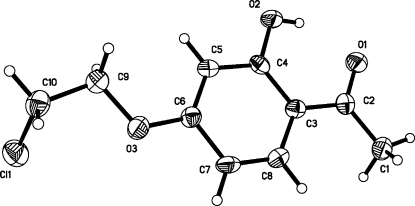
The molecular structure of (I), with atom labels and displacement ellipsoids drawn at the 30% probability level.

## supplementary crystallographic information

### Comment

In this paper, we used the Williamson reaction (Dermer, 1934) to form the title compound, (I), which was synthesized by the reaction of 2,4-dihydroxyacetophenone, potassium carbonate and 1-bromo-2-chloroethane at 329 K. In (I)(Fig.1), the bond lengths and angles are normal and comparable to those observed in the related structure (Schlemper, 1986). The dihedral angle between the benzene ring C3—C8 and the plane O3C9C10 is 5.83 (4)°. There are no significantly short intermolecular contacts in the crystal lattice.

### Experimental

2, 4-Dihydroxylacetonephenone (5 mmol), potassium carbonate (6 mmol), 1-bromo-2-chloroethane (5 mmol), and 50 ml acetone were mixed in 100 ml flask. After 2.5 h stirring at 329 K, the crude product was obtained. The crystals suitable for X-ray analysis were obtained by slow evaporation of a solution of the title compound in n-hexane/ethyl acetate/methanol (3:3:1, V/V) at 283 K.

### Refinement

The positions of all H atoms were fixed geometrically and distance to H atoms were set by the program, with C—H distance in the range 0.93–0.97 Å and O—H distance of 0.82 Å, and refined as riding, with *U*_iso_(H)= 1.2–1.5*U*_eq_(C,*O*).

### Crystal data

**Table:**

C_10_H_11_ClO_3_	*D*_x_ = 1.442 Mg m^−^^3^
*M**_r_* = 214.64	Melting point = 375–376 K
Orthorhombic, *P**c**a*2_1_	Mo *K*α radiation, λ = 0.71073 Å
*a* = 8.9970 (7) Å	Cell parameters from 1122 reflections
*b* = 5.3258 (4) Å	θ = 3.8–21.7°
*c* = 20.6307 (17) Å	µ = 0.36 mm^−^^1^
*V* = 988.55 (13) Å^3^	*T* = 298 K
*Z* = 4	Orthorhombic, colourless
*F*(000) = 448	0.40 × 0.39 × 0.20 mm

### Data collection

**Table:**

Siemens SMART CCD area-detector diffractometer	1664 independent reflections
Radiation source: fine-focus sealed tube	1121 reflections with *I* > 2σ(*I*)
graphite	*R*_int_ = 0.063
phi and ω scans	θ_max_ = 25.0°, θ_min_ = 3.8°
Absorption correction: multi-scan (*SADABS*; Sheldrick, 1996)	*h* = −10→8
*T*_min_ = 0.868, *T*_max_ = 0.931	*k* = −6→6
4434 measured reflections	*l* = −24→20

### Refinement

**Table:**

Refinement on *F*^2^	Secondary atom site location: difference Fourier map
Least-squares matrix: full	Hydrogen site location: inferred from neighbouring sites
*R*[*F*^2^ > 2σ(*F*^2^)] = 0.052	H-atom parameters constrained
*wR*(*F*^2^) = 0.141	*w* = 1/[σ^2^(*F*_o_^2^) + (0.0604*P*)^2^ + 0.0181*P*] where *P* = (*F*_o_^2^ + 2*F*_c_^2^)/3
*S* = 1.07	(Δ/σ)_max_ < 0.001
1664 reflections	Δρ_max_ = 0.24 e Å^−^^3^
127 parameters	Δρ_min_ = −0.21 e Å^−^^3^
1 restraint	Absolute structure: Flack (1983), 758 Friedel pairs
Primary atom site location: structure-invariant direct methods	Flack parameter: 0.10 (14)

### Special details

**Table:**

Geometry. All e.s.d.'s (except the e.s.d. in the dihedral angle between two l.s. planes) are estimated using the full covariance matrix. The cell e.s.d.'s are taken into account individually in the estimation of e.s.d.'s in distances, angles and torsion angles; correlations between e.s.d.'s in cell parameters are only used when they are defined by crystal symmetry. An approximate (isotropic) treatment of cell e.s.d.'s is used for estimating e.s.d.'s involving l.s. planes.
Refinement. Refinement of *F*^2^ against ALL reflections. The weighted *R*-factor *wR* and goodness of fit *S* are based on *F*^2^, conventional *R*-factors *R* are based on *F*, with *F* set to zero for negative *F*^2^. The threshold expression of *F*^2^ > σ(*F*^2^) is used only for calculating *R*-factors(gt) *etc*. and is not relevant to the choice of reflections for refinement. *R*-factors based on *F*^2^ are statistically about twice as large as those based on *F*, and *R*- factors based on ALL data will be even larger.

### Fractional atomic coordinates and isotropic or equivalent isotropic displacement parameters (Å^2^)

**Table:**

	*x*	*y*	*z*	*U*_iso_*/*U*_eq_	
Cl1	−0.15098 (17)	0.8043 (3)	0.62958 (8)	0.0704 (5)	
O1	0.2660 (4)	0.6840 (7)	1.02143 (16)	0.0554 (10)	
O2	0.1105 (4)	0.4294 (7)	0.94300 (15)	0.0518 (9)	
H2	0.1390	0.4778	0.9786	0.078*	
O3	0.1071 (4)	0.6890 (7)	0.72354 (16)	0.0560 (10)	
C1	0.4075 (6)	1.0330 (9)	0.9931 (3)	0.0535 (14)	
H1A	0.4253	1.0346	1.0390	0.080*	
H1B	0.3628	1.1890	0.9803	0.080*	
H1C	0.5000	1.0116	0.9706	0.080*	
C2	0.3071 (5)	0.8247 (9)	0.9769 (2)	0.0399 (11)	
C3	0.2555 (5)	0.7861 (9)	0.9118 (2)	0.0387 (11)	
C4	0.1582 (5)	0.5869 (9)	0.8976 (2)	0.0384 (11)	
C5	0.1058 (5)	0.5501 (9)	0.8351 (2)	0.0414 (12)	
H5	0.0405	0.4190	0.8265	0.050*	
C6	0.1495 (5)	0.7049 (9)	0.7863 (2)	0.0401 (12)	
C7	0.2485 (6)	0.9076 (9)	0.7991 (3)	0.0480 (13)	
H7	0.2794	1.0143	0.7660	0.058*	
C8	0.2962 (5)	0.9404 (9)	0.8603 (2)	0.0451 (13)	
H8	0.3599	1.0739	0.8687	0.054*	
C9	0.0041 (6)	0.4949 (9)	0.7074 (2)	0.0518 (13)	
H9A	−0.0826	0.5050	0.7352	0.062*	
H9B	0.0499	0.3316	0.7133	0.062*	
C10	−0.0399 (6)	0.5287 (9)	0.6389 (3)	0.0571 (14)	
H10A	−0.0957	0.3831	0.6245	0.068*	
H10B	0.0484	0.5425	0.6122	0.068*	

### Atomic displacement parameters (Å^2^)

**Table:**

	*U*^11^	*U*^22^	*U*^33^	*U*^12^	*U*^13^	*U*^23^
Cl1	0.0739 (9)	0.0668 (9)	0.0706 (9)	0.0068 (7)	−0.0081 (8)	0.0050 (10)
O1	0.065 (2)	0.055 (2)	0.046 (2)	−0.009 (2)	−0.0029 (19)	0.0086 (19)
O2	0.062 (2)	0.046 (2)	0.0474 (19)	−0.0151 (18)	−0.0008 (16)	0.0123 (17)
O3	0.061 (2)	0.063 (2)	0.044 (2)	−0.0216 (19)	−0.0055 (16)	0.0038 (19)
C1	0.049 (3)	0.051 (3)	0.061 (3)	0.002 (3)	−0.008 (3)	−0.007 (3)
C2	0.032 (2)	0.044 (3)	0.044 (3)	0.010 (2)	0.003 (2)	−0.004 (3)
C3	0.033 (2)	0.039 (3)	0.044 (3)	0.001 (2)	0.007 (2)	0.003 (2)
C4	0.039 (3)	0.038 (3)	0.039 (3)	0.009 (2)	0.003 (2)	0.006 (2)
C5	0.046 (3)	0.032 (3)	0.046 (3)	−0.003 (2)	0.001 (2)	0.003 (2)
C6	0.045 (3)	0.039 (3)	0.036 (3)	−0.001 (2)	0.002 (2)	−0.005 (2)
C7	0.050 (3)	0.038 (3)	0.056 (3)	−0.011 (2)	0.007 (3)	0.011 (3)
C8	0.043 (3)	0.040 (3)	0.052 (3)	−0.008 (2)	0.000 (2)	−0.002 (2)
C9	0.053 (3)	0.048 (3)	0.055 (3)	−0.003 (3)	−0.005 (3)	−0.001 (2)
C10	0.062 (3)	0.055 (3)	0.055 (3)	0.002 (3)	−0.001 (3)	−0.014 (3)

### Geometric parameters (Å, °)

**Table:**

Cl1—C10	1.787 (5)	C4—C5	1.387 (7)
O1—C2	1.242 (6)	C5—C6	1.359 (6)
O2—C4	1.329 (5)	C5—H5	0.9300
O2—H2	0.8200	C6—C7	1.425 (6)
O3—C6	1.352 (6)	C7—C8	1.344 (7)
O3—C9	1.428 (6)	C7—H7	0.9300
C1—C2	1.469 (7)	C8—H8	0.9300
C1—H1A	0.9600	C9—C10	1.477 (8)
C1—H1B	0.9600	C9—H9A	0.9700
C1—H1C	0.9600	C9—H9B	0.9700
C2—C3	1.436 (7)	C10—H10A	0.9700
C3—C8	1.392 (7)	C10—H10B	0.9700
C3—C4	1.407 (7)		
			
C4—O2—H2	109.5	O3—C6—C7	113.7 (4)
C6—O3—C9	116.9 (4)	C5—C6—C7	120.2 (4)
C2—C1—H1A	109.5	C8—C7—C6	118.2 (5)
C2—C1—H1B	109.5	C8—C7—H7	120.9
H1A—C1—H1B	109.5	C6—C7—H7	120.9
C2—C1—H1C	109.5	C7—C8—C3	123.8 (5)
H1A—C1—H1C	109.5	C7—C8—H8	118.1
H1B—C1—H1C	109.5	C3—C8—H8	118.1
O1—C2—C3	120.6 (4)	O3—C9—C10	108.0 (4)
O1—C2—C1	118.1 (4)	O3—C9—H9A	110.1
C3—C2—C1	121.3 (5)	C10—C9—H9A	110.1
C8—C3—C4	116.7 (4)	O3—C9—H9B	110.1
C8—C3—C2	123.0 (5)	C10—C9—H9B	110.1
C4—C3—C2	120.3 (4)	H9A—C9—H9B	108.4
O2—C4—C5	117.2 (4)	C9—C10—Cl1	110.7 (4)
O2—C4—C3	122.0 (4)	C9—C10—H10A	109.5
C5—C4—C3	120.8 (4)	Cl1—C10—H10A	109.5
C6—C5—C4	120.3 (4)	C9—C10—H10B	109.5
C6—C5—H5	119.8	Cl1—C10—H10B	109.5
C4—C5—H5	119.8	H10A—C10—H10B	108.1
O3—C6—C5	126.1 (4)		
			
O1—C2—C3—C8	179.0 (4)	C9—O3—C6—C7	−178.1 (4)
C1—C2—C3—C8	0.0 (7)	C4—C5—C6—O3	179.8 (4)
O1—C2—C3—C4	−0.4 (7)	C4—C5—C6—C7	−0.5 (7)
C1—C2—C3—C4	−179.3 (4)	O3—C6—C7—C8	179.5 (4)
C8—C3—C4—O2	−179.2 (4)	C5—C6—C7—C8	−0.2 (7)
C2—C3—C4—O2	0.2 (6)	C6—C7—C8—C3	0.7 (7)
C8—C3—C4—C5	−0.4 (6)	C4—C3—C8—C7	−0.4 (7)
C2—C3—C4—C5	179.0 (4)	C2—C3—C8—C7	−179.7 (4)
O2—C4—C5—C6	179.7 (4)	C6—O3—C9—C10	173.7 (4)
C3—C4—C5—C6	0.8 (7)	O3—C9—C10—Cl1	−69.2 (5)
C9—O3—C6—C5	1.6 (7)		

### Hydrogen-bond geometry (Å, °)

**Table:**

*D*—H···*A*	*D*—H	H···*A*	*D*···*A*	*D*—H···*A*
O2—H2···O1	0.82	1.81	2.533 (5)	145
